# Asthma: Differential Diagnosis and Comorbidities

**DOI:** 10.3389/fped.2018.00276

**Published:** 2018-10-03

**Authors:** Nicola Ullmann, Virginia Mirra, Antonio Di Marco, Martino Pavone, Federica Porcaro, Valentina Negro, Alessandro Onofri, Renato Cutrera

**Affiliations:** Paediatric Pulmonology & Respiratory Intermediate Care Unit, Sleep and Long Term Ventilation Unit, Department of Paediatrics, Paediatric Hospital “Bambino Gesù” Research Institute, Rome, Italy

**Keywords:** differential diagnosis, asthma, diagnosis, comorbidities, wheeze, asthma mimics

## Abstract

Childhood asthma remains a multifactorial disease with heterogeneous clinical phenotype and complex genetic inheritance. The primary aim of asthma management is to achieve control of symptoms, in order to reduce the risk of future exacerbations and progressive loss of lung function, which results especially challenging in patients with difficult asthma. When asthma does not respond to maintenance treatment, firstly, the correct diagnosis needs to be confirmed and other diagnosis, such as cystic fibrosis, primary ciliary dyskinesia, immunodeficiency conditions or airway and vascular malformations need to be excluded. If control remains poor after diagnostic confirmation, detailed assessments of the reasons for asthma being difficult-to-control are needed. Moreover, all possible risk factors or comorbidities (gastroesophageal reflux, rhinosinusitis, dysfunctional breathing and/or vocal cord dysfunction, obstructive sleep apnea and obesity) should be investigated. At the same time, the possible reasons for poor symptom control need to be find in all modifiable factors which need to be carefully assessed. Non-adherence to medication or inadequate inhalation technique, persistent environmental exposures and psychosocial factors are, currently, recognized as the more common modifiable factors. Based on these premises, investigation and management of asthma require specialist multidisciplinary expertise and a systematic approach to characterizing patients' asthma phenotypes and delivering individualized care. Moreover, since early wheezers are at higher risk of developing asthma, we speculate that precocious interventions aimed at early diagnosis and prevention of modifiable factors might affect the age at onset of wheezing, reduce the prevalence of persistent later asthma and determine long term benefits for lung health.

## Introduction

Childhood asthma is a multifactorial disease with heterogeneous clinical phenotype and complex genetic inheritance. The primary aim of asthma management is to make an early diagnosis and to achieve a prompt control of symptoms, in order to reduce the risk of future exacerbations and progressive loss of lung function (1). When asthma is correctly diagnosed, low-dose inhaled corticosteroids can easily control symptoms in most of patients. When asthma does not respond to usual treatment and higher maintenance doses are needed or not sufficient, we enter in the “problematic severe asthma” field (2, 3). However, in some cases, we would probably better define it as “problematic respiratory symptoms unresponsive to prescribed asthma therapy” (4). Nowadays, all pediatricians feel probably confident in clinically diagnose a child with asthma and functional respiratory tests, as a simple spirometry, are often not even performed before starting the basic treatment. Pediatric asthma increased in numbers due to a greater awareness, however, lately we moved from the problem of under diagnosing to a possibly worse condition of over diagnosing asthma (4). The known expression: “Not all that wheezes is asthma” continues to be relevant. In front of a child with ongoing symptoms despite correct treatment, the first consideration is to ensure if the diagnosis of asthma is in fact correct. Depending on the clinical signs and symptoms, alternative diagnosis needs to be taken into consideration, a new correct diagnosis might be made and a different management followed. Differently, when the detailed re-evaluation confirms the diagnosis of asthma, all possible risk factors or comorbidities need to be considered and, sometimes treated, to ensure the maximal effort to obtain symptoms control. At the same time, doctors should verify that basic asthma management strategies are in place. Often the poor symptom control is a consequence of modifiable factors which need to be carefully assessed such as: (1) non-adherence to medication, (2) inadequate inhalation technique, (3) persistent environmental exposures and (4) psychosocial factors. Anytime our asthma management is not satisfying and we categorize our patient as “difficult to treat asthmatic,” we need to remember that the first step is always to go right back to square one, instead of keep increasing the corticosteroid treatment: the differential diagnosis, with the most positive attitude of looking for our possible own “mistakes.”

The main aim of this paper is to offer a clinical overview of all possible asthma mimickers which need to be taken into consideration and of the comorbidities that might contribute at the poor symptoms control.

## Problematic severe asthma or problematic respiratory symptoms

As respiratory pediatricians we all experienced that low-dose inhaled corticosteroids can easily control asthma symptoms in most of patients. Moreover, exacerbations in children are often concentrated in some periods of the year due to viral trigger or aeroallergens, and some little patients can even stop their treatment in summer time without clinical deterioration. Nevertheless, children and their parents sometimes keep complaining of recurrent respiratory symptoms despite the correct and continuous treatment, and various courses of antibiotics, beta-2 agonists and oral corticosteroids are frequently prescribed by pediatrician. As a consequence of the poor symptoms control, asthma treatment is often increased and the patient categorized as “problematic severe asthmatic.” However, an earlier step could be identified and this condition better defined as “problematic respiratory symptoms unresponsive to prescribed asthma therapy” (4). In fact, the known expression: “Not all that wheezes is asthma” continues to be relevant and the asthma diagnosis should be clinically reconsidered. In the last few decades, we possibly moved from under diagnosing to over diagnosing asthma. Nowadays, a thriving child with history of recurrent cough and a need of frequent beta-2 and inhaled corticosteroid (ICS) treatment is often diagnosed with “probable asthma” and ICS in ever increasing doses immediately prescribed. The initial suspected diagnosis, made with insufficient features and no spirometry testing, gradually, and with no evidence, becomes a definitive diagnosis rarely questioned in the future. Over diagnosis of asthma is a significant problem both in primary and secondary care (5, 6). With this new concept, before an unjustified referral for difficult asthma is made, all the components of the airway disease need to be reconsidered and atypical signs or symptoms which point to an alternate diagnosis recognized. “False asthmas” are often diagnosed years after the onset of clinical symptoms, leading to excessive use of anti-asthmatic medications other than dangerous progression of the misdiagnosed chronic primary disease (7). As often suggested, the first step is to go back to the beginning and take a really detailed history and perform a complete physical examination.

## The history and physical examination

Asthma diagnosis is often suspected when a constellation of typical features is present, however, as most guidelines highlight, diagnosis has to be confirmed either by objective tests or an objective response to treatment. One of the initial major error that occurs is that insufficient features of the asthma pattern are sought before the diagnosis is made. Patient's history should always give enough answers to the following question: “Does the child have any or all of cough, wheeze and breathlessness?.” A persistent cough (especially if non-productive) alone in an otherwise well-child is highly unlikely be due to asthma (8). The medical term wheezing is often used by parents to describe different sounds and care should be taken to be sure what they actually mean (9, 10). The habit to ask the family to try to demonstrate the sound of wheezing yourself, could help to avoid misunderstanding. It is also important to understand if the child is noisy and breathless at the same time and to assess the severity of symptoms. After an intense exercise, breathlessness is common but a formal diagnosis of asthma or exercise-induced laryngeal obstruction (EILO) is correct only in about half of those complaining of symptoms. The rest of adolescents are just deconditioned or exaggerating their symptoms (11). The possibility of fabricated and induced illness should be also excluded and possible personal or family psychological factors should be investigated. If symptoms are not clear and an uncontrolled symptomatic asthma need to be ruled out, instead of an extra increase of ICS or of a useless beta-2 prescription, an exercise test may be useful. One of the most “typical” asthma symptoms, such as cough is often present but, at the same time, is definitively a non-specific symptom. In fact, a cough may be the first symptom of many diseases or conditions affecting the respiratory tract. Almost all diseases of the respiratory tract, and in some cases of the extra-respiratory tract, can cause cough (12).

If symptoms are referred just during daytime (no cough or wheezing at night or first hours of morning) and with a sudden onset of symptoms in the absence of obvious triggers, asthma cannot be definitively excluded but other possible diagnosis should be seriously considered. Finally, pattern of symptoms need to be explored with important implications for therapy. For example, of particular importance is distinguishing children only with asthma induced by viral symptoms from those with elapsing symptoms between colds, the latter being more compatible with a chronic disease. At this stage, the atopic status of the family and of the patient should already being stated or it would be necessary to do so, in fact, non-atopic pediatric severe asthma exists but it is rare.

Moreover, history should always include specific questions on parents' smoking habits, pets and home environment. Information on pregnancy, delivery and neonatal period could lead our attention to rule out different diseases: persistent symptoms from birth or first weeks of life are rarely due to an asthmatic condition (13).

A careful physical examination is as important as a careful history. Examination is usually normal, however atypical signs and symptoms need to be identified to think of alternative possible diseases. The diagnosis of asthma should be reconsidered in case of the following: (1) presence of clubbing, cyanosis, significant anemia or nasal polyps; (2) failure to thrive (13).

The presence of symptoms “typical” of asthma plus the finding on physical examination of wheezing strongly point to a diagnosis of asthma. Confirmation of the diagnosis of asthma is based on three key additional elements:

- The demonstration of variable expiratory airflow limitation, preferably by spirometry, when possible.
- Documentation of reversible obstruction.
- Exclusion of alternative diagnoses (see “Alternative Diagnosis” below).

Evidence of airway obstruction on spirometry, especially if reversible with a bronchodilator, can confirm the diagnosis of asthma or assess the severity (14, 15). However, normal spirometry does not exclude the diagnosis. Moreover, young or non-compliant patients are often unable to perform. Improvement on a trial of medications is often used to confirm the diagnosis in these patients (16).

Measurements of peak expiratory flow using a peak flow meter are more variable and effort dependent. Thus, peak flow measurements alone should not be used to diagnose asthma but is more often used in the follow-up of patients to monitor patients' symptoms and response to therapy.

Since, especially in young patients, there is no gold standard test for asthma diagnosis, other possible tests could be performed such as: allergy testing, done either by skin or *in vitro*. Outdoor aeroallergens are unusual triggers in infants and very young children but may be triggers in older children. In addition, when indicated testing reveals the presence of IgE antibody to any allergen, an atopic diathesis is demonstrated, increasing the likelihood that chest symptoms are due to asthma.

In older children bronchoprovocation testing (with methacholine, cold air, or exercise) could be performed when the clinical features are suggestive of asthma but spirometry is normal and there is no response to asthma medications. An exercise challenge of sufficient magnitude may provoke symptoms in children with asthma (17). A negative bronchoprovocation study may also be useful in reducing the likelihood that a child has asthma, although it cannot be used to exclude the diagnosis. Chest radiograph should be performed only in children whit persistent symptoms who do not respond to treatment. In those children, the chest radiograph may display findings suggestive of causes for wheezing other than asthma. Exhaled nitric oxide testing is not recommended as a diagnostic tool but is mostly used in the follow up of asthmatic patients.

## Alternative diagnosis

### When to look for other diagnosis

As specified above, the persistent condition of “problematic respiratory symptoms unresponsive to prescribed asthma therapy” need to motivate a clinical full reassessment to exclude that the patient was wrongly labeled with the diagnosis of asthma. To be able to do so, all possible conditions which might mimic asthma need to be considered. Some diseases, better defined as comorbidities, might also coexist with a diagnosis of asthma and will be discussed in a specific section.

Table 1 modified from British Guideline on the Management of Asthma, enlists the clinical clues to alternative diagnosis in asthmatic young children (18). Table 2 lists alternative diagnosis for “persistent respiratory symptoms not responding to asthma treatment” and the main clinical characteristics for each.

**Table 1:** Clinical clues to alternative diagnosis in children with wheezing.

**Clinical clue**	**Possible diagnosis**
**PERINATAL AND FAMILY HISTORY**	
Symptoms present from birth	Chronic lung disease of prematurity, PCD, CF
Family history of unusual chest disease	CF, Neuromuscular disorders, PCD
Severe upper respiratory tract disease	PCD
**SYMPTOMS AND SIGNS**	
Persistent moist cough	PBB, Bronchiectasis, Recurrent aspiration, PCD, CF
Excessive vomiting	GERD (w/without aspiration)
Dysphagia	Swallowing problems (w/without aspiration)
Breathlessness with light headedness and peripheral tingling	Dysfunctional breathing, Panic attacks
Inspiratory stridor	Tracheal or laryngeal disorder
Abnormal voice or cry	Laryngeal problems
Focal signs in chest	Developmental anomaly, FB, Post-infective syndrome
Persistent wheeze	Extrinsic intra thoracic airway compression, Airway-malacia, Luminal obstruction, CF, FB
Finger clubbing	CF, Bronchiectasis
Failure to thrive	CF, GERD

*CF, cystic fibrosis; FB, foreign body; GERD, gastro-esophageal reflux disease; PBB, protracted bacterial bronchitis; PCD, primary ciliary dyskinesia*.

**Table 2:** When to suspect specific alternative diagnosis and initial useful diagnostic examinations.

**Alternative diagnosis**	**When to suspect**	**Useful diagnostic examinations**
Cystic fibrosis and bronchiectasis	Daily cough productive of sputum, clubbing, malabsorption and failure to thrive, recurrent chest infections, airways bacterial colonization	Sweat chloride test, Genetic tests, Swab culture, Lung Function tests, Chest CT
Immunodeficiency	Recurrent airway infections, Systemic infections (from a few months of age)	Immunoglobulins and specific tests
Primary ciliary dyskinesia	Neonatal upper airway symptoms, Chronic rhinosinusitis, Recurrent otitis media, Daily wet cough, Laterality defects	Nasal NO, HSVM, EM, Genetic tests, Immunofluorescence, Chest CT
Protracted Bacterial Bronchitis	Cronich wet cough, Poor response to Beta-2 agonists, Good response to prolonged course of antibiotics	Often no need of examinations, Swab culture, Bronchoscopy with BAL
Airway malacia	Monophonic wheeze when the child is active, High risk setting (i.e., pt operated for tracheo-esophageal fistula or vascular ring), Presence of associated stridor	Lung function test (truncated expiratory flow in spirometry), Flexible bronchoscopy, Dynamic CT
Airway foreign body	Abrupt onset of symptoms, History of choking, Unilateral monophonic wheeze, Focal hyperinflation of lung	Bronchoscopy, chest x-ray
Habit cough	Prolonged dry, honking cough; Absence of cough during sleep; Absence of any physical findings	Medical investigations should be avoided
Vocal cord dysfunction	Absence of structural abnormalities, Sudden worsening of “asthma” symptoms, No response to asthma medications	Video of an attack, Laryngoscopy during attack
Bronchiolitis obliterans	History of severe viral respiratory infection in the first 3 years of life	CT scan (characteristic mosaic pattern and air trapping)

*CT, computed tomography; EM, eletcron microscopy; HSVM, high speed video microscopy; NO, nitric oxide*.

Symptoms and clinical signs present from birth, such as: daily productive cough, recurrent upper and lower airway infections could suggest Cystic Fibrosis, Primary Ciliary Dyskinesia or an Immunodeficiency condition. When a chronic wet cough responds to prolonged antibiotic treatment a diagnosis of Protracted Bacterial Bronchitis should be made. Inspiratory sounds or difficult breathing suggest extra-thoracic pathological conditions (tracheal or laryngeal disorders). Respiratory problems combined with significant gastresophageal symptoms should prompt investigations to rule out: GER, recurrent aspiration, swallowing problems. Moreover, the sudden onset of respiratory symptoms with mono lateral thoracic sounds could be indicative for airway foreign body.

Finally, doctors should also remember that a child who presented early on his life one or few episodes of preschool wheeze or dry cough related to colds or upper airway infections should not be labeled with a diagnosis of asthma. Many of these patients in fact are just “healthy” children that will stop experiencing wheezing and respiratory symptoms at school age. This correct medical attitude will possibly avoid overtreatment, medicalization, and anxiety in parents.

### Cystic fibrosis, non-CF bronchiectasis and immunodeficiency

One of the typical symptoms of a patient affected by Cystic Fibrosis (CF) or diffuse non-CF bronchiectasis is chronic cough with the production of abundant sputum which is definitively atypical for a severe asthmatic child. Moreover, CF patients suffer from recurrent chest infections from a very young age and might present episodes of bronchial asthma, which could lead to some diagnostic confusion (19–22).

In non-CF bronchiectasis, the most frequently reported symptoms are productive cough; sputum expectoration; respiratory distress; growth retardation; night sweats (23, 24).

Similarly, respiratory symptoms and complications present a significant cause of morbidity and also mortality among patients suffering from different forms of Primary immunodeficiencies. Frequent and severe infections of either upper (e.g., sinusitis and otitis media) or lower respiratory tract (e.g., pneumonia, bronchitis, bronchiectasis, and interstitial lung diseases) are typical of an immunodeficiency condition. (25–27). Failure to thrive signs and sign of malabsorption could be observed both in CF and immunodeficiency patients.

### Primary ciliary dyskinesia

Lower airways are commonly involved in Primary Ciliary Dyskinesia (PCD). One of the most common manifestation is chronic asthma, that is generally unresponsive to maintenance treatment, especially at school-age and during adolescence (28). A mild to moderate obstructive pattern is frequently found at spirometry. Possible pathological changes accounting for this include airway smooth muscle hypertrophy and fibrosis, intraluminal secretions and altered lung mechanics secondary to repeated infection (29). Frequently, heterogeneous clinical presentation asthma symptoms let to a challenging diagnosis. More worrying, many patients remain not diagnosed until adulthood when they might already have developed lung bronchiectasis. Lower and upper recurrent respiratory symptoms with no obvious other cause, such as prematurity should suggest to rule out this diagnosis (30). Later in childhood, these children suffer of chronic moist cough often triggered by viral infections, which is one of the reason for years of misdiagnosis with bronchial asthma.

A history of unexplained respiratory distress at birth, recurrent otitis, lower and upper respiratory infections require further examinations. Nasal nitric oxide (nNO) measurement represents a helpful screening tool for PCD diagnosis. nNO levels are extremely low in PCD compared to healthy and disease controls (31). Otherwise, there is no single gold standard diagnostic test for PCD. A complete diagnostic work-up, including high-speed video microscopy analysis, transmission electron microscopy, immunofluorescence, sinus and chest TC is mandatory when PCD is highly suspected (32).

The management of PCD patients involves mainly airway clearance, infection control, and elimination of exposure to inflammatory triggers, also including passive smoke, without which asthma could not improve.

### Protracted bacterial bronchitis

In children with chronic cough (lasting ≥4 weeks) not responding to asthma treatment, protracted bacterial bronchitis (PBB) should be clinically suspected and a prolonged (minimum of 2 weeks) course of antibiotic should be prescribed. PBB is often misdiagnosed as asthma, resulting in inappropriate and often high doses of inhaled Corticosteroids. Children with PBB were usually young and scarce systemic symptoms, without evidence of sinusitis or ear disease. They typically appeared well, with normal growth and development (33, 34).

Untreated, this condition over time can lead to suppurative lung disease and bronchiectasis. Lung function tests are usually normal (35).

### Airway malacia

Medical awareness for this condition is unfortunately still low and a good number of children are still misdiagnosed as asthmatic after several years of fruitless asthma treatment. (36). Malacic airways (laryngomalacia, tracheomalacia, bronchomalacia) are the most common congenital abnormalities of the pediatric airway and are characterized by excessive softness of tissues leading to increased airway compliance and excessive dynamic collapse during the respiratory cycle (37). Laryngomalacia can be suspected when positional stridor occurs in infants. Even though it is usually a benign condition resolving spontaneously, symptom persistence associated to feeding disturbance, poor weight gain, chest retraction and progressive deformation of rib cage require other investigation to exclude neurologic, genetic and cardiac disorders. When malacia involves intra thoracic airways barking cough, diffuse (tracheomalacia) or unilateral (bronchomalacia) monophonic wheeze, and prolonged expiratory phase expiratory symptoms may occur and must be distinguished from asthma. Sometimes, excessive dynamic collapse can also cause cyanotic spells, apnea and difficulty weaning ventilator support (38).

Congenital tracheomalacia may occur in isolation but has also been associated with other airway abnormalities (tracheoesophageal fistulas, laryngeal clefts, laryngomalacia, and bronchomalacia). Differently, acquired tracheomalacia occurs in the normally developed trachea that undergoes damage from external compression (tumors, cysts, goiter, vascular structures), trauma (tracheostomy), positive pressure ventilation, infection, or inflammation (38). The specific characteristic cough sound often triggered after viral respiratory infection and the flow-volume spirometry pattern (reduction in peak expiratory flow) can help to suspect this condition (39). Other more invasive tests are needed for final diagnosis, such as flexible bronchoscopy and dynamic CT scan with contrast enhancement (40). Observation and conservative management are typically all that are required. However, surgical intervention can be necessary in the most severe cases, and can result in significant improvement in symptoms (37).

### Airway foreign body

Usually, the sudden onset of respiratory symptoms is more suggestive for a diagnosis of an accidental aspiration of foreign body than of a sudden onset of asthma. Definitively, it is more common in young children (<4 years old). History could be positive for choking and immediate distress but the absence of such history does not certainly rule out the diagnosis. Clinically patient might present persistent cough, unilateral and monophonic wheeze and respiratory distress. However, symptoms depend on the grade of airway obstruction (complete or partial).

### Habit cough

Diagnosis can usually be suspected when the patient coughs repeatedly during the visit, especially when the cough is noising and referred exclusively during daily hours. Usually these children would have received many therapies, including asthma drugs, with no benefit on the honking, brassy cough of many weeks duration, often occurring after a respiratory tract infection. It is more common in children older than 8 years and can cause undue distress to children and both parents. The cough disappears as soon as the child is distracted and always absent during sleep. The origins of the habit cough are often obscure, in some patients can also occur in the background of mild asthma. The diagnosis is made after exclusion of other causes. Some patients might respond to control of breathing exercises from a physiotherapist or speech therapist. Psychological stresses need to be sought in the child's school or home life. The help of a psychologist may be needed to identify and resolve the problem.

### Psychological issues

There is a growing evidence in literature that stress could be a mediator of atopy and that worsens asthma in children. On one hand, asthma control shall be reduced in stressed patients and families due to a poor adherence to treatment; on the other hand, neuroimmunological mechanisms may influence asthma when a psychological distress occurs (41). Lind et al. found that stress, exhaustion, anxiety, depression, were higher than normal in allergic asthma and atopic dermatitis, but not in nonallergic asthma. The vicious circle between psychological issues and symptoms is well-established: inflammatory and non-inflammatory mechanisms contribute to enhance atopy and asthma symptoms (42, 43). Finally, has been demonstrated the strong relationship between panic disorder and asthma. The typical presentation of panic disorder is the hyperventilation: rapid expiration causes dehydration of the airway contributing to induce asthma by airway narrowing (44).

### Vocal cord dysfunction

This condition is characterized by inappropriate movement of the vocal cords (adduction during inspiration) induced by: (a) exercise (b) psychological stress (c) local irritation–i.e., reflux. Often vocal cord dysfunction coexists with asthma but it is unresponsive to short-acting beta-2 agonists and it presents with intermittent inspiratory symptoms. The diagnosis of vocal cord dysfunction involves an accurate medical history and specific exams. Patients report air hunger, intermittent aphonia or dysphonia, sensation of choking, chest tightness, chest pain, difficulty swallowing, fatigue and throat clearing. Endoscopic examination with direct visualization of the vocal cords via flexible, transnasal fiber-optic laryngoscopy, possibly after bronchial challenge or during an acute attack, is the gold standard for the diagnosis. Spirometry may show an abnormal shape of inspiratory loop consistent with a variable extrathoracic obstruction (45). Treatment depends on the underlying cause, however, often children are taught breathing control exercises, encouraging nose breathing, and appropriate use of the diaphragm (46). This intervention is often leaded by a respiratory physiotherapist or speech therapist and may help resolving the problem (47–49).

### Bronchiolitis obliterans

Bronchiolitis obliterans (BO) is frequently secondary to influenza, parainfluenza, measles, respiratory syncytial virus, varicella, and Mycoplasma pneumoniae. However, adenovirus is by far the most common agent linked to the development of BO, and can present with persistent wheezing, rather than paroxysmal symptoms. CT scan will show a characteristic mosaic pattern and air trapping (50).

## Asthma plus: co-morbidities

In cases where symptoms remain uncontrolled despite maximal guideline-recommended treatment, and all alternate diagnosis have been excluded, possible comorbidities need to be investigated as they may be a coincidental finding or they may contribute directly for the severity of asthma (51).

Establishing the real impact and the causative effect of comorbidities on asthma control it is complicated, and a medical treatment is sometimes necessary to assess their role (52). In most cases, although the ability to improve pediatric severe asthma by treating comorbidities remains unconfirmed, a therapeutic trial should be prescribed.

### Obesity

Obesity is a risk factor for poor asthma control, but the relationship between the two conditions is still under debate. It is evident that breathlessness due to simple deconditioning may lead to an erroneous diagnosis of asthma, however, there might be some interesting recent findings. Both, overweight and deconditioning merit treatment in their own right, however weight reduction is known to be very difficult to achieve. Recently, it was found an increased prevalence of asthma in obese children and this group of patients showed more severe exacerbations (greater risk of admission to pediatric intensive care unit) suggesting that asthma in obesity could be a specific disease (53). Moreover, obesity seems to be possible associated with steroid resistance (54). However, we need to be cautious to consider a real association between severe asthma and obesity, as results might be confounded by the known association of obesity with low socio-economic conditions and all what this brings (55). Finally, obesity is known to be associated with variable increased inflammatory phenotypes and airway may be the target of systemic inflammation (56). Obese asthmatic patients probably will also need to be investigated for obstructive sleep apneas and treated appropriately (57). In conclusion, behavioral and weight reduction programs should be offered to asthmatic obese patients to obtain weight reduction. At the same time, it is important to assess airway disease and, before steroid therapy is escalated, look for evidence of uncontrolled airway inflammation (4).

### Rhinosinusitis

Pediatric chronic rhinosinusitis (CRS) represent different stages of one chronic inflammatory disease of the mucosa of the nasal cavity and paranasal sinuses, resulting from repeated acute bacterial rhinosinusitis, and leading to a “maladaptive-eosinophilic” stage disease. CRS is a major disease condition with high morbidity and can influence lower airway disease status in adults. In a cross-sectional Korean study, including 17,506 participants, CRS was significantly related to asthma, in particular CRS without nasal polyps was related to childhood-onset asthma (onset before 18 years) or early-onset asthma (onset before 40 years) in adults (58). Given this potential evolution toward more irreversible disease, a more aggressive early intervention it is hoped, to prevent these long-term consequences. Coexistence of chronic rhinosinusitis with nasal polyps and asthma and rather similar characteristics of inflammation support assumption that chronic rhinosinusitis and nasal polyps and asthma may be, at least in part, the same disease process. Chronic rhinosinusitis with nasal polyps is estimated to occur in 7% of all asthmatics, whereas asthma is reported to occur in 26–48% of patients with Chronic rhinosinusitis with nasal polyps (59). Patients with allergic rhinitis and/or chronic rhinosinusitis report poorer asthma control, more exacerbations and emergency visits and have more difficulty achieving symptom control (60), and increased asthma severity has also been shown to be associated with sinonasal inflammation (61). Upper airway symptoms can significantly affect patients' quality of life and should be treated irrespectively of any benefit on asthma control, which could also be obtained (62). A study by Penn et al. examined the efficacy of anti-IgE therapy for the treatment of Chronic rhinosinusitis with nasal polyps. Despite the small number of patients, the nasal polyp scores significantly improved in the anti-IgE group (63, 64). In a recent randomized, double-blind, placebo-controlled trial, Gevaert et al. reported a reduction of nasal polyp size and an improvement of symptoms, compared with placebo in patients with Chronic rhinosinusitis with nasal polyps independent of atopic status (65). Otherwise, despite these promising results, further studies are needed in order to confirm them, as well as of other biologics.

### Gastro-esophageal reflux

Symptoms of GER often coexist in children with severe asthma. Micro-aspiration, acid stimulation of the esophagus and vagus nerve stimulation are the mechanism proposed to explain how the GER can trigger asthma (66). Furthermore, GER can trigger VCD with consequent laryngeal dysfunction mimicking asthma symptoms. Anyway, it has been proven that GER treatment does not improve asthma control (67). This may suggest that symptoms triggered by GER are mimics of asthma symptoms, rather than exacerbating airway inflammation or airway hyperresponsiveness. Specific examinations assessing for GER, such as impedance-pHmetry and/or gastroesophageal endoscopy must be request in order to exclude this comorbidity (68). A trial of treatment with PPI is recommended as the initial diagnostic step in symptomatic children (69).

### Food allergy

It is still debated whether there is a real relationship between difficult asthma and food allergy, however, there is a higher incidence of food allergy in asthmatic patients admitted to intensive care units and thus it could be considered as a comorbid condition (70). In the literature evidences that support the close relationship between food allergy and asthma as well as the early food sensitization or allergy preceding the development of asthma and other atopic disease can be found (71).

Sometimes patients with food allergy report bronchospasm caused by ingestion or inhalation of the offending food. Even though it is a rare condition (72) linked to an underlying bronchial reactivity, it may develop either in children suffering from asthma or in non-asthmatic patients as part of an allergic reaction to food. When the ingestion of the offending food occurs closely to physical exertion, asthma and anaphylactic symptoms may develop configuring the clinical picture of food-dependent exercise-induced anaphylaxis (73).

It is already showed that symptomatic sensitization to foods is associated with asthma (74) and that the risk of asthma morbidity (daytime symptoms, hospital admissions, lower percent predicted forced expiratory volume in 1-s, asthma persistence, severe asthma exacerbation) is higher in children with food allergy. This risk is especially increased in children with high levels of food specific IgEs and multiple or severe food allergy (75, 76).

When food allergy is diagnosed (suggestive clinical history, positive skin prick tests and specific IgE, positive oral food challenge), the avoidance of the offending food should be suggested, even though specific immunotherapy can be also considered in selected cases to alter the course of atopic march (77).

## Problematic asthma or true severe uncontrolled asthma

During the previous process of considering alternative diagnosis and the possible presence of comorbidities that may impact asthma clinical expression and control in childhood, doctors should always verify that basic asthma management strategies are in place (78). Often, the poor symptom control is a consequence of modifiable factors which need to be carefully assessed before proceeding to more invasive investigations. The most frequent conditions that might justify a poor control of symptoms are: non-adherence to medication, inadequate inhalation technique, persistent environmental exposures and psychosocial factors (79). Finally, all patients and carers must be given an individualized written asthma action plan that details current treatment, how to recognize and treat an exacerbation and when to seek appropriate help (47).

## Summary and conclusions

In conclusion, since it is known that the majority of asthmatic children can be well-controlled with the basic treatment, if a patient keeps complaining of severe or recurrent respiratory symptoms despite all the basic asthma management strategies are in place, alternative diagnosis need to be considered. Any deviation from common typical patterns should alert one to keep a high index of suspicion for diseases that mimic asthma. Moreover, all possible co-morbidities that might contribute to the uncontrolled clinical condition, need to be excluded and eventually treated. To do so, we need to remember that there is no substitute for a good clinical evaluation that can be performed by all doctors before an unnecessary referral is made.

## Author contributions

NU, VM, and FP drafted the initial manuscript, searched for bibliography and revised the final manuscript. VN, AD, and MP were involved in drafting the manuscript, critically revised the manuscript, and approved the final manuscript. RC and NU made substantial contributions to conception and design of the study. RC reviewed and approved the final manuscript. All authors read and approved the final manuscript as submitted.

### Conflict of interest statement

The authors declare that the research was conducted in the absence of any commercial or financial relationships that could be construed as a potential conflict of interest.
